# Reuse of phosphogypsum pretreated with water washing as aggregate for cemented backfill

**DOI:** 10.1038/s41598-022-20318-0

**Published:** 2022-09-27

**Authors:** Yanan Zhou, Xibing Li, Ying Shi, Quanqi Zhu, Jing Du

**Affiliations:** 1 School of Resources and Safety Engineering, Central South University, 932 Lushan South Rd, Changsha, 410083 China; 2 School of Materials Engineering, Changshu Institute of Technology, 99 South Third Ring Rd, Changshu, 215500 China

**Keywords:** Environmental impact, Mineralogy

## Abstract

Phosphogypsum (PG) is reused as aggregate in the cemented backfill, which effectively improves the PG reutilization efficiency. However, the massive impurities contained in aggregate PG would adversely affect the hydration of binder, and therefore deteriorate the strength development of backfill. This research starts with the feasibility study on pretreating PG with the water washing method. Based on the most economical principle of the water demand, the optimal conditions for washing PG were determined at a stirring time of 5 min and a solid–liquid ratio of 1:0.5. Then, the original and pretreated PG were made into the backfill. Compared to using the original PG, the backfill slurry using the pretreated PG had better fluidity performance, such as the lower slurry viscosity and the higher bleeding rate. Furthermore, with the pretreated aggregate PG, the backfill strength was significantly enhanced by more than 8 times. Finally, the environmental behavior of the cemented backfill was investigated. Using the pretreated PG as aggregate, concentrations of PO_4_^3−^ and F^−^ in the bleeding water and backfill leachates could meet the Chinese standard for integrated wastewater discharge. The results extend the reuse of PG as aggregate in a more environmental-friendly way, meeting the needs for sustainable mines.

## Introduction

Cemented backfill is an effective means to increase ore recovery, improve safety conditions, and reduce surface disposal of solid wastes. As a typical solid waste, phosphogypsum (PG) is the by-product generated during the exploitation of phosphate resources^1–3^. Global production of PG is estimated to be around 100–280 Mt annually, of which China contributes to 25%^4,5^. Currently, PG is recycled as additives in building materials, soil modifiers and cement productions, but with a limited utilization rate of 15%^6–8^. In 2008, Li et al.^9^ innovatively proposed a cemented backfill technique with PG as aggregate, effectively improving PG utilization rate up to 60%. In the cemented PG backfill process, the aggregate PG (over 80% by dry weight) is mixed with binder and water to a heterogeneous backfill slurry, which is then pumped to the underground mined-out areas. The slurry gradually dewaters and consolidates, building up strength to support the rock walls in the underground mines.

As the primary backfill material, aggregate PG is mainly composed of CaSO_4_·2H_2_O, and it also contains large quantities of impurities such as residual acids, phosphates, fluorides and heavy metals^10,11^. Previous studies have shown that the impurities might seriously deteriorate the hydration process of the backfill and cause serve environmental pollution. Li and Fall^12^ added sulfate in slag-cemented paste backfill and found that high sulfate content negatively impacted the early age strength and self-desiccation of the backfill. Chen et al.^13^ explored the effects of chloride on the mechanical properties of gangue-cemented paste backfill. The results showed that the early strength of backfill decreased obviously when the initial chlorine content was more than 40‰. Zhou et al.^14^ prepared cemented backfill using PG with various phosphate contents and demonstrated that the 120d strength decreased from 2.04 to 0.30 MPa as the dissolved phosphate in PG increased from 29 to 377 mmol/kg. When the phosphate content in PG exceeded 87 mmol/kg, it tends to cause phosphate pollution to the environment. Furthermore, it is worth noting that PG is a hyperacidic solid waste with a pH value usually within 3, compared with other neutral filling aggregates^1,15^. However, hydration reactions commonly occur under strongly alkaline conditions (pH > 11.5)^16^. Therefore, the residual acids in PG would neutralize hydroxyl ions of binder and interfere with the hydration reaction of the backfill, which in turn disturbs the strength development of the backfill. As a result, it is necessary to pretreat PG to mitigate the adverse effects in their secondary utilization.

Actually, several studies have found that the pretreatment of solid waste can effectively improve the workability of cementation and reduce environmental pollution. Singh^17^ depicted that the pretreated PG (treating with 3–4% aqueous citric acid) could be used as an additive in place of mineral gypsum for the manufacture of ordinary Portland cement and Portland slag cement. Mao et al.^18^ washed fly ash with water, and it was found that the consolidation rate of heavy metals was above 92% in the treated fly ash. When it was prepared as cementing materials, the consolidation rate was further increased to over 99%, resulting in the leaching concentration of heavy metals was far lower than the national standard limit. Based on these results, the pretreatment of aggregate PG should be considered to reduce the impurities content, therefore ensuring safety for the mining and environment.

The PG pretreatment protocols are currently main as follows: chemical, thermal and physical treatments^19–21^. Chemical and thermal treatments of PG can effectively reduce soluble impurities and organic matters, but the operation process is cumbersome and costly. Generally, the physical treatment of PG, especially water washing, is still preferred in the industry due to its simple operation. Singh et al.^22^ washed PG at a volume proportion of 1:3 for three durations of 30, 50 and 65 min, and found that 63.0% of phosphates, 66.1% of fluorides and 80.7% of organic matters could be removed. Subsequently, Zhao et al. ^23^ washed PG with a mass ratio of PG to water of 1:10 for 30 min, and the results showed a reduction in soluble phosphates from 0.79 to 0.46%, fluorides from 0.87 to 0.61% and magnesium from 0.09 to 0%, respectively. These findings indicate that impurities can be reduced by washing PG with varying solid–liquid (S/L) ratios and stirring times. Although the water washing method has been studied for decades, most of the previous studies usually washed PG for only one time to calculate washing efficiency. However, the actual washing process is influenced by multiple factors^18,24^. Moreover, PG is a slightly water-soluble substance containing a variety of impurities, and the process of water washing PG must be complicated. Therefore, it is essential to determine the optimal condition of water washing that are beneficial for the properties of backfill and friendly to the environment.

The purpose of this study is to further explore the effect of pretreatment aggregate on the cemented backfill process. By considering different stirring durations, the number of washing times and the S/L ratios, the optimal condition of water washing pretreatment of PG was determined. Following this, the original PG with different initial pH values was collected as the control group. Subsequently, the original and pretreated PG were made into cemented backfill. The properties of the backfill slurry, the strength and microstructure of hardened backfills, and the resultant surrounding environment impacts were investigated.

## Experimental method

### Materials

This study evaluated representative samples of PG and composite binder in Guizhou, China. The binder is composed of yellow phosphorous slag: fly ash: cement clinker in 4:1:1, and 16–20% lime of the yellow phosphorous slag mass ratio is added. The main chemical compositions (measured by X-ray fluorescence; Bruker, Switzerland) and physical properties (measured by a particle size analyzer; Malvern Instruments, UK)of PG with different pH values were investigated through the toxicity leaching test, as listed in Table 1.

**Table 1:** Impurity concentrations and physical properties of PG and binder.

Impurity concentration	Raw PG					Binder
	PG-1	PG-2	PG-3	PG-4	PG-5	
pH	1.75	1.99	2.63	3.52	4.99	13.16
PO_4_^3−^ (mg/L)	4840	4000	1510	252	20	0.04
F^−^ (mg/L)	1641	1103	521	509	182	16
SO_4_^2−^ (mg/L)	10,885	10,042	4419	2313	1359	487
TDS (ppt)	7.89	3.55	2.79	1.63	0.73	4.91
**Physical property**						
D_10_ (µm)	13.56	11.94	11.94	10.61	15.61	6.02
D_30_ (µm)	42.79	29.18	33.15	36.10	37.76	13.73
D_60_ (µm)	92.05	55.24	81.02	67.08	81.31	30.18
C_u_ = D_60_/D_10_	6.79	4.63	6.79	4.04	5.21	5.01
C_c_ = D_30_^2^/(D_60_*D_10_)	1.47	1.29	1.14	1.17	1.12	1.04

### Water washing design and cemented backfill procedures

In this study, different methods were used to wash the PG. Due to the strong acidity of PG-1 with an initial pH value of 1.75, which was selected to study the effects of stirring time and S/L ratio on the water washing of PG. In the test on the effect of stirring time on PG, the wet mass of PG was weighted according to Table 2. Then, PG and deionized water with the S/L ratio of 1:2 were mixed thoroughly with a stirrer at a speed of 200 rpm/min. The homogeneous mixture was taken out at 1, 2, 5, 10, 30, 60, 120 and 240 min, respectively. Subsequently, the mixture was centrifuged at 4000 r/min for 15 min. After centrifugation, the supernatant was collected to measure the pH value and the concentrations of PO_4_^3−^, F^−^, and SO_4_^2−^. In the test on the effect of S/L ratio on PG, four ratios were used, ranging from 1:0.5 to 1:2. Then, the PG was weighed before mixing with deionized water of a certain ratio and centrifuged after stirring for 5 min. In the test on the effect of initial pH values on PG, the PG with initial pH values of 1.75, 1.99 and 2.63 (PG-1, PG-2 and PG-3) were selected. The PG was weighed before mixing with deionized water at the S/L ratio of 1:0.5 and a stirring time of 5 min. Then, the homogeneous mixture was centrifuged and the supernatant was collected for the following measurement. The weight of dry PG, wet PG, water for the first washing and each subsequent washing is shown in Table 2.

**Table 2:** Mix design of water washing pretreatment of PG samples.

PG No	S/L ratio	Weight of PG		Weight of water	
		Dry (g)	Wet (g)	First washing (g)	Next washing (g)
PG-1	1:0.5	100	149.10	0.90	50.00
	1:1	100	149.10	50.90	100.00
	1:1.5	100	149.10	100.90	150.00
	1:2	100	149.10	150.90	200.00
PG-2	1:0.5	100	123.44	26.56	50.00
	1:1	100	123.44	76.56	100.00
	1:1.5	100	123.44	126.56	150.00
	1:2	100	123.44	176.56	200.00
PG-3	1:0.5	100	100.33	49.67	50.00
	1:1	100	100.33	99.67	100.00
	1:1.5	100	100.33	149.67	150.00
	1:2	100	100.33	199.67	200.00

**Figure 1:**
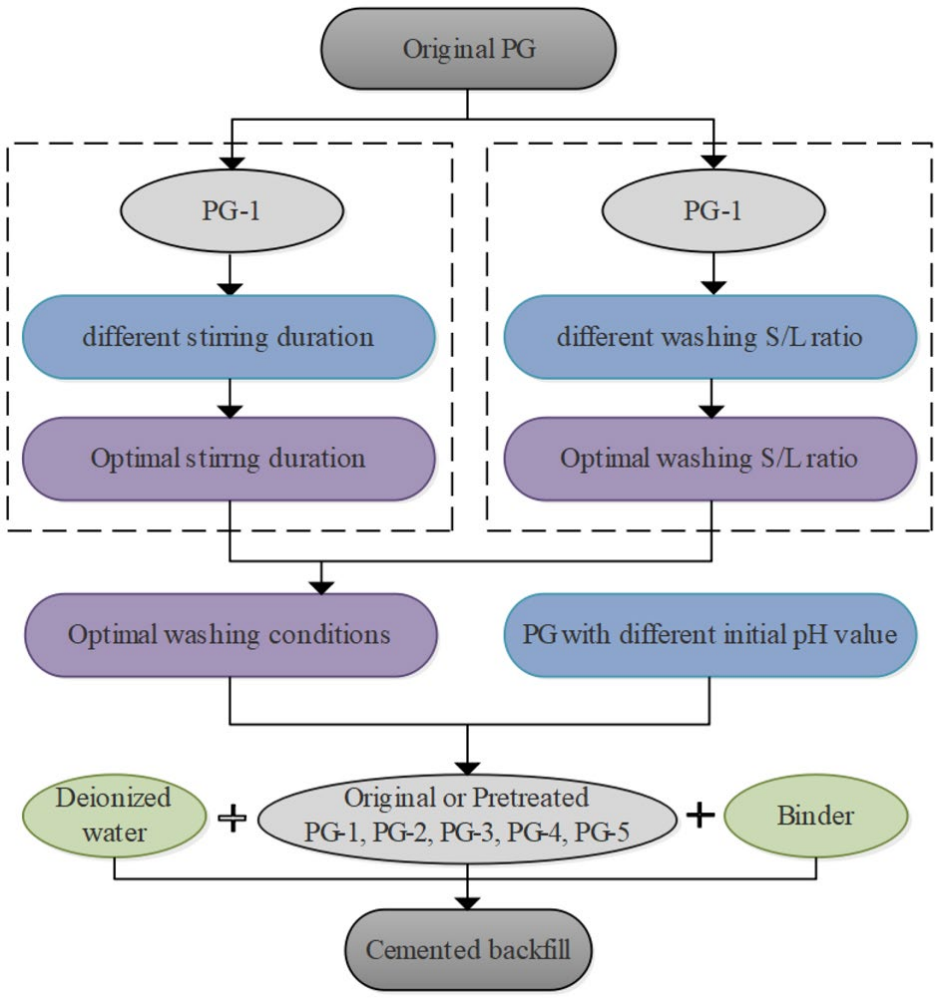
Flow diagram of the experiment.

Backfill slurry was prepared by mixing the PG, binder and deionized water with a mass proportion of 5:1:6. In accordance with the experiment scheme, PG and deionized water were first mixed evenly in the stirred vessel to prevent blocking. Then the binder was slowly poured into the PG mixture and stirred homogeneously at 200 rpm/min for 30 min. Next, the backfill slurry was injected into plastic molds with internal dimensions of 40 × 40 × 40 mm. There was a 0.2 mm small hole at the bottom of the mold to drain the excess water in the slurry. After the slurry was set, the hardened samples were taken out of the molds and cured into a chamber maintained with a constant temperature of 20 ± 2 °C and humidity of 90 ± 5%. The flow diagram for this work is shown in Fig. 1.

### Test methods

#### Bleeding rate

The bleeding rate was measured according to the Chinese standard GB/T 50080-2016. The backfill slurry was injected into a container with a lid and then placed on a vibrator for 20 s to make the slurry denser. The bleeding water was drawn with a syringe at every 30 min until no more water was secreted for three consecutive times. The bleeding rate was calculated by using Eq. (1):

1 $$B = \frac{Vw}{{\left( \frac{W}{G} \right)Gw}} \times 100$$

where *B* is the bleeding rate (%), *Vw* is the mass of bleeding water in the container (g), *W* is the total mass of water in the backfill slurry (g), *G* is the total mass of backfill slurry (g), *Gw* is the mass of backfill slurry in the container (g).

#### Apparent viscosity

The apparent viscosity is one of the essential rheological properties of backfill slurry^25^, which affects a series of actual conditions such as slurry transportation and pumping. The apparent viscosity of slurry was evaluated according to ASTM D2196-18 by using a DV-1 digital viscometer (Brookfield, USA). Due to the continuous hydration reaction of the slurry, in order to ensure the reliability of the test data, the measurement should be conducted immediately after slurry preparation.

#### Setting time

The initial setting time (IST) and final setting time (FST) of the backfill slurry were determined according to the Chinese standard GB/T 1346-2011 with a Viac apparatus. The prepared backfill slurry was first poured into a Vicat mold, and then the mold was gently shaken several times to scrape off the excess slurry. Finally, measuring and recording IST and FST at regular intervals with a Vicat needle.

#### Uniaxial compressive strength

Uniaxial compressive strength (UCS) is an effective and straightforward method to evaluate the quality of backfill. According to Chinese standard JGJ/T 70-2009, the UCS tests were carried out on the cemented backfill samples cured for 28d with a displacement rate of 0.1 mm/min using a servo-hydraulic machine (Hualong, China). Three samples were used for each UCS test, and the average values were calculated.

#### Microstructural analysis

The scanning electron microscope (SEM) analysis was carried out with HELIOS NamoLab 600i (FEI, USA) to analyze the microstructure and element types of PG and backfill samples. After UCS tests, the broken samples were immediately placed into the anhydrous ethanol solution to prevent hydration reaction. Then the samples were dried at 40 °C in a drying oven until a constant weight was obtained. Due to the inferior conductivity of PG and backfill samples, the surface of the samples was coated with gold (Au) for 240 s to satisfy the conductivity requirements.

#### Toxicity leaching test and chemical analysis

In order to investigate the concentration of impurities in PG and backfill samples, the toxicity leaching test was conducted according to HJ 557-2010. After curing for 28d, the backfill samples were grounded and sieved through a 3.0 mm screen. The powders were mixed with deionized water in a container at a mass proportion of 1:10 and shaken at 110 rpm/min on a rotary shaker for 8 h. Then the mixtures were placed on the table for 16 h. Finally, the mixtures were filtered through a 0.45 mm filter, and the leachates were collected for further analysis.

The pH value of PG, bleeding water and the leachate of toxicity leaching test was measured by pH meter (Ohaus, US). The concentrations of SO_4_^2−^ and PO_4_^3−^ were determined by ammonium molybdate tetrahydrate spectrophotometry (Shimadzu, Japan). The total dissolved solids (TDS) and the concentration of F^−^ were measured by TDS meter (Ohaus, US) and fluorine ion-selective electrode (Leici, China), respectively.

## Results and discussion

### Effect of water washing conditions on PG

Due to the production processes and stockpile environment, different quantities of impurities are contained in PG. Meanwhile, the types of impurities are also affected by residual acids in PG^26,27^. In this study, the pH value is used as an index to evaluate washing efficiency. The following studies aim to provide an optimal stirring time and S/L ratio for the actual water washing of PG.

#### Effect of stirring time on PG

**Figure 2:**
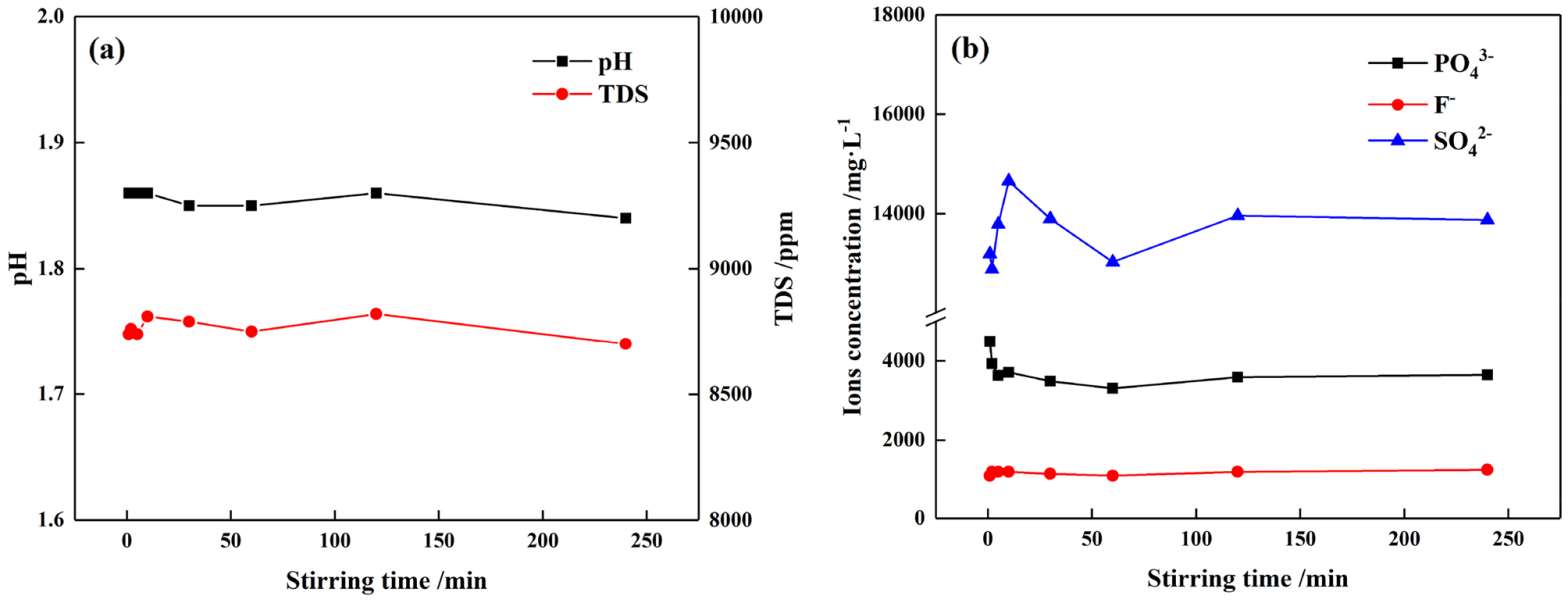
Variations of PG with different stirring times: (**a**) pH value and TDS, (**b**) concentrations of PO_4_^3−^, F^−^ and SO_4_^2−^.

PG and deionized water with a mass ratio of 1:2 were mixed thoroughly, and the mixture was taken out at 1, 2, 5, 10, 30, 60, 120 and 240 min, respectively. The pH value and the concentrations of PO_4_^3−^, F^−^, and SO_4_^2−^ in the mixture are shown in Fig. 2.

As presented in Fig. 2a, with the first 1 min of stirring, the pH value of PG reached 1.86 and remained stable afterward. The increase of pH value was mainly due to the residual acids absorbed on the surface of the PG crystals, which were easily detached from the PG surface and escaped into the solution during the stirring process. In addition, it can be seen from Fig. 2b that the concentration of impurities showed evident changes within the 5 min. This undulation of impurities concentrations was due to complicated chemical reactions occurring in the PG solution, such as dissolution and recrystallization of CaSO_4_·2H_2_O, the ion-exchanges of PO_4_^3−^, F^−^, and SO_4_^2−^^28^. Then, the impurities reached an equilibrium state after 5 min, and the concentrations of PO_4_^3−^, F^−^ and SO_4_^2−^ stabilized at about 3500 mg/L, 1200 mg/L and 14,000 mg/L, respectively. The TDS also remained at about 8800 ppm within 5 min (seen in Fig. 2a), which indicated that the dissolved ions absorbed on the PG surface had been well diffused into the solution. In general, it can be inferred that the optimal stirring time for water washing PG is 5 min in this study.

#### Effect of solid–liquid ratio on PG

The water demand affects the labor and material resources that an enterprise needs to invest. In the actual water washing process, the S/L ratio may be directly related to the water demand. In this study, the water demand is defined as the ratio of water consumed for washing PG to the dry weight of PG. Therefore, PG was washed with different S/L ratios of 1:0.5, 1:1, 1:1.5 and 1:2 for 5 min each time until the pH reached a pre-designated value. According to the previous research results and the accumulated experiences, when the pH value of PG is about 5.00, it has little influence on the cemented PG backfill technique^29,30^.

**Figure 3:**
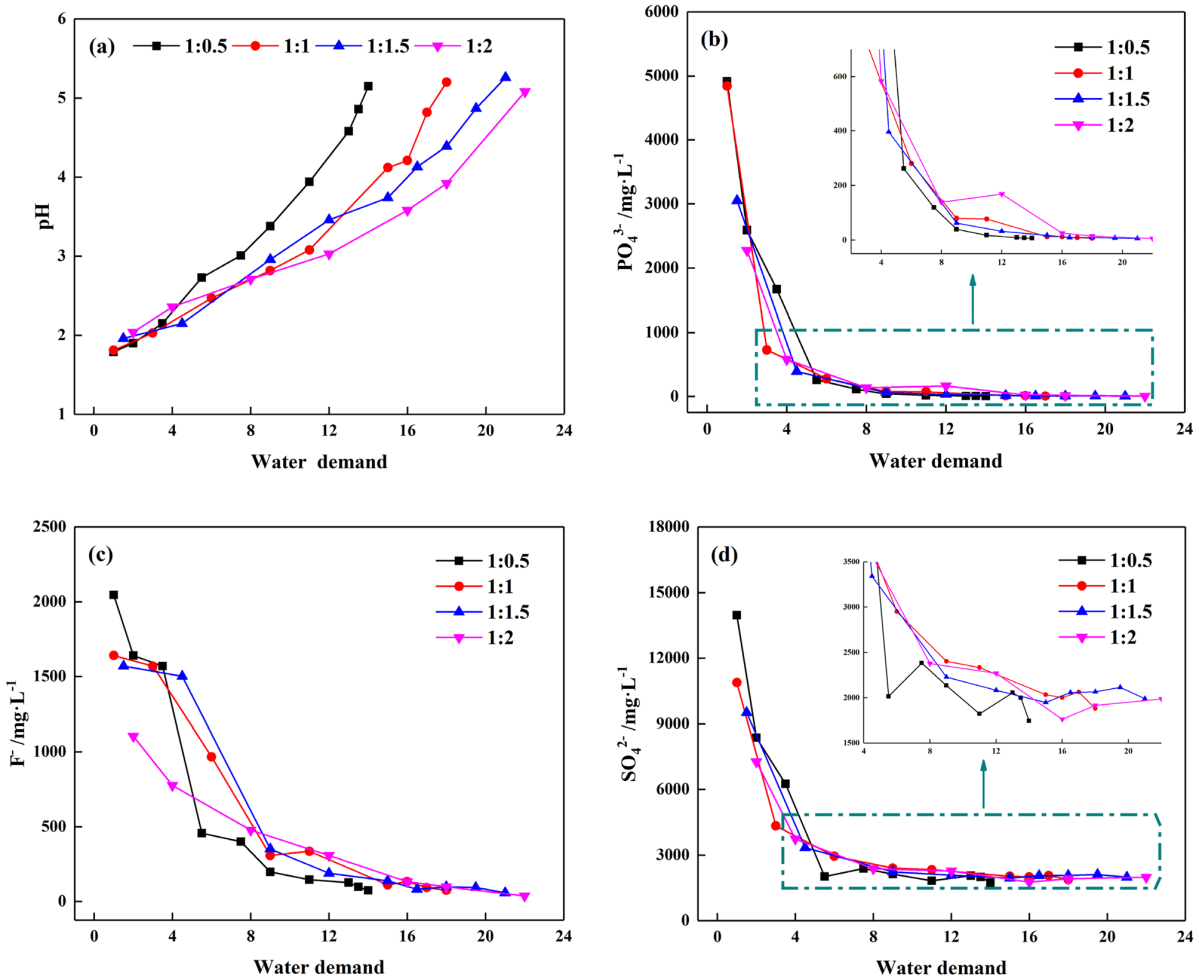
Variations of PG with different S/L ratio: (**a**) pH, (**b**) PO_4_^3−^, (**c**) F^−^, (**d**) SO_4_^2−^.

As clearly shown in Fig. 3a, the pH value increased along with the water demand. The gradual growth of pH value was owing to the removal of residual acids by water washing. With a pH value of 5.00 as the target, washing PG with the S/L ratio of 1:0.5 required a water demand of 14. While the water demand of washing PG with the S/L ratio of 1:1, 1:1.5 and 1:2 was1.3, 1.5, and 1.6 times than that of 1:0.5, respectively. As regards the changes in impurity concentrations during water washing, Fig. 3b–d present the variation curves of PO_4_^3−^, F^−^ and SO_4_^2−^ concentrations with water demand. It can be seen that the concentrations of all impurities decreased dramatically in the early washing times, and over 80% of impurities were removed at the first 8 washing demands. Then the pace of changes gradually slowed down. It was worth noting that with the S/L ratio increased from 1:0.5 to 1:2, the removal efficiencies of PO_4_^3−^, F^−^ and SO_4_^2−^ changed slightly. According to the above results, it can be inferred that when the PG is washed multiple times with a lower S/L ratio, a more rapid increase in the pH value of PG and a more significant reduction in the impurities concentration can be achieved with the minimum water demand. Therefore, the S/L ratio of 1:0.5 can be considered as the optimal ratio with acceptable efficiency in this study.

**Figure 4:**
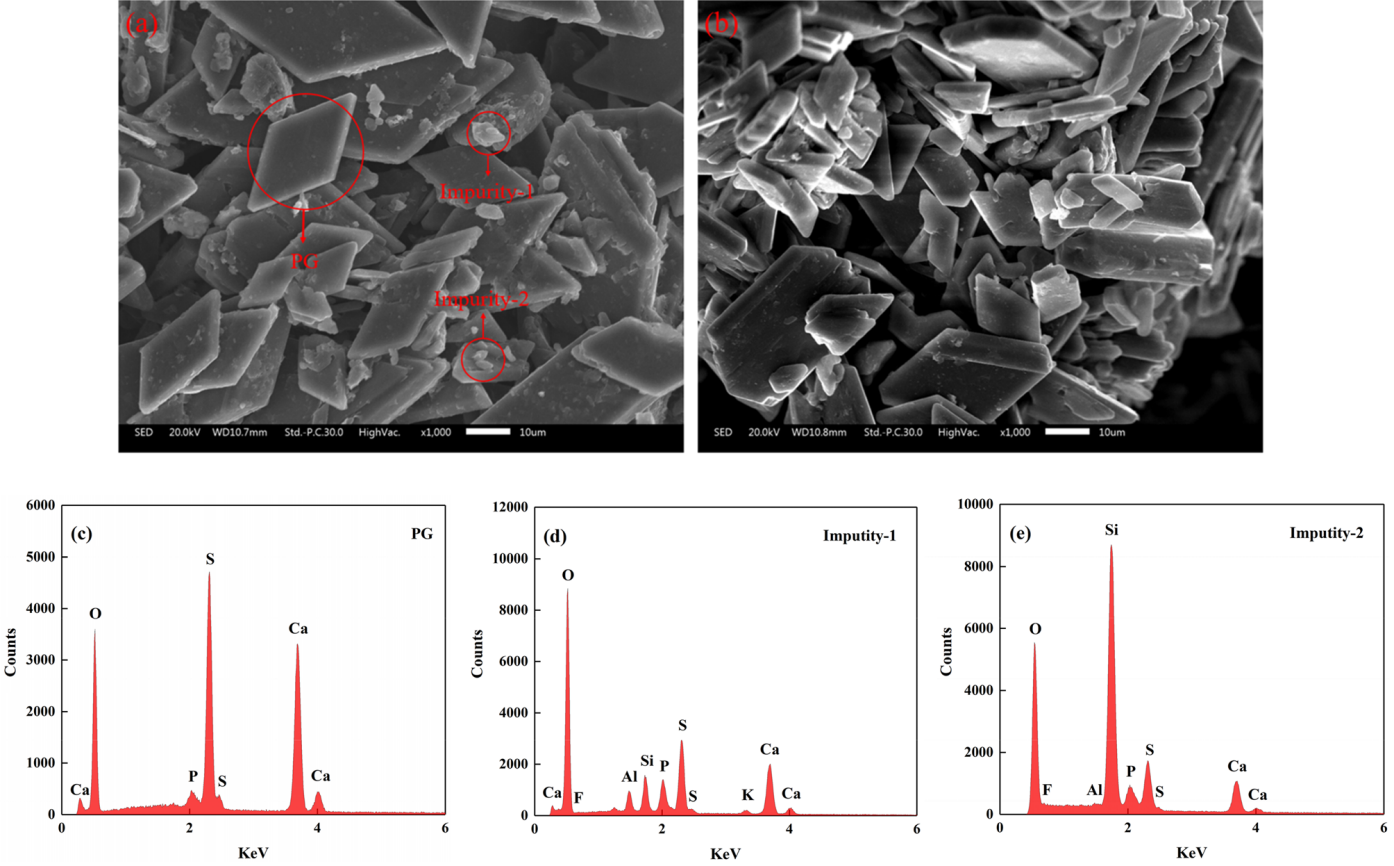
SEM images of PG: (**a**) original PG-1 with a pH value of 1.75, (**b**) pretreated PG-1 with a pH value of 5.15, (**c**) EDS of PG, (**d**) EDS of impurity-1, (**e**) EDS of impurity-2.

The morphological structure of the PG with and without pretreatment was observed by SEM analysis. Figure 4a is the SEM image of the original PG with a pH value of 1.75, and Fig. 4b is the SEM image of pretreated PG with a pH value of 5.15. It is well known that the PG crystals are plate-like structures^31^. Obviously, large quantities of small irregular particles were absorbed on the surface of PG crystals that could be directly identified in Fig. 4a. In comparison, as shown in Fig. 4b, water washing did not change the structure of PG crystals. However, the amount of irregular particles initially attached on the PG crystals significantly reduced, and the surface became smooth. To further understand the composition of irregular particles, EDS analysis was performed. The results showed that massive Ca, O and S were detected in the irregular particles, and a certain amount of F, P, K, Al, and Si were also measured (seen in Fig. 4d, e). Therefore, it is considered that these small particles attached to the PG surface might be impurity particles. The SEM images also confirm that water washing could effectively remove the impurities.

#### Effect of initial pH value of PG

**Figure 5:**
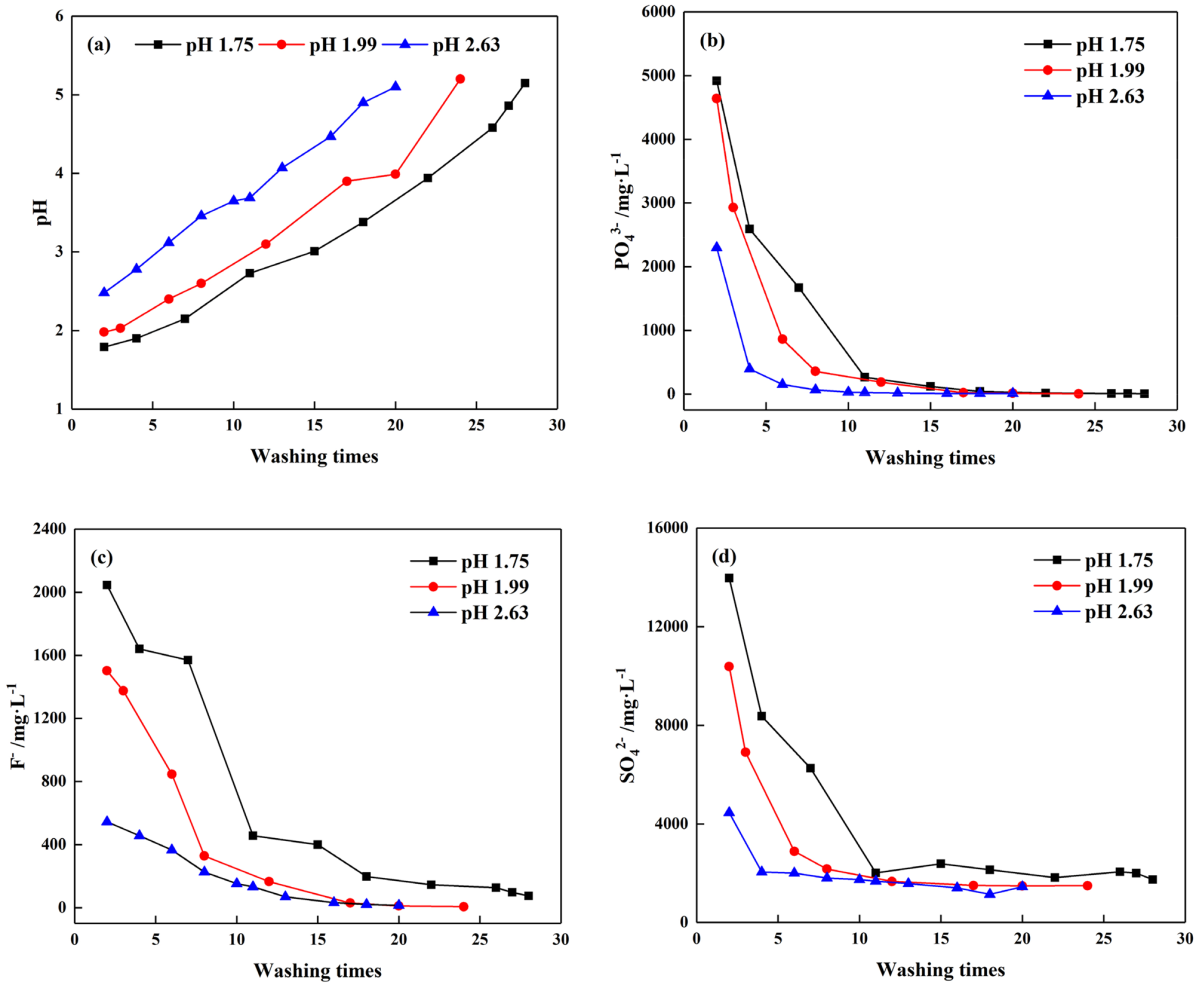
Variations of pretreated PG with different initial pH value: (**a**) pH, (**b**) PO_4_^3−^, (**c**) F^−^, (**d**) SO_4_^2−^.

Three batches of PG (PG-1, PG-2 and PG-3) with an initial pH value of 1.75, 1.99, and 2.63 were selected to study the effect of water washing on the initial pH value of PG. The PG was washed by an S/L ratio of 1:0.5 and a stirring time of 5 min as determined from the above tests until the pH value of PG was 5.00. As presented in Fig. 5a, for PG with initial pH of 2.63, the pH was raised to 3.00 only by washing 6 times. And after 20 times of washing, the pH value was higher than 5.00. However, for the original PG with initial pH of 1.75 and 1.99, 28 and 24 times of washing were needed to raise the pH to 5.00. It is evident that PG with a lower pH value contained more H^+^, and more water was needed to remove the acidity and raise the pH value of PG. Thus, PG with a lower initial pH value needs more washing times to reach the specified pH value.

Regarding the impurity concentrations in the original PG, it can be seen that the higher the initial pH of PG, the fewer impurities were observed. This might be attributed to that some impurities were removed under the different stockpiles environments and weathering factors, resulting in fewer impurities contents in the original PG ^10^. The effect of washing times on PO_4_^3−^, F^−^ and SO_4_^2−^ concentration are shown in Fig. 5b–d, respectively. The impurities concentration dropped rapidly before washing 10 times, meaning that excessive soluble impurities on the PG surface can be easily dissolved in the liquid. However, the descending rate gradually decreased in the following washes, leading to a corresponding decrease in the removal efficiency. Compared to PG-1 and PG-2, PG-3 needed fewer washing times to remove the impurities. The 5% difference in removal efficiency between two adjacent washes is defined as the stabilization of PG in this study. As seen in Fig. 5b, the concentration of PO_4_^3−^ in PG-3 was stabilized only by 6 washing times. While for PG-1 and PG-2, 15 and 8 washing times were needed, respectively. For F^−^, more washes were needed to stabilize, and it was 16 washes for PG-3, which had the lowest initial F^−^ content. It indicated that F^−^ would be released continuously in PG in the long run. Eventually, when PG were all washed to the pH value of 5.00, the impurities in the washing solution was about varied from 5 to 8 mg/L of PO_4_^3−^, 6 to 75 mg/L of F^−^ and 1400 to 1750 mg/L of SO_4_^2−^. This result infers that even after multiple washing times, the concentrations of F^−^ and SO_4_^2−^in PG remain high, posing potential environmental hazards if without further treatment.

### Backfill slurry properties of purified PG

In order to investigate the influence of pretreated PG on the properties of backfill slurry, PG-1, PG-2 and PG-3 were washed to pH values of 3.50 and 5.00, respectively. The washing conditions are based on the most economical principle of the water demand determined by the above tests (the optimal washing S/L ratio of 1:0.5 and washing time of 5 min). In addition, PG-4 and PG-5 with an initial pH of 3.52 and 4.99 were selected as control groups. The experimental results of viscosity, bleeding rate and setting times (IST and FST) are presented in Table 3.

**Table 3:** Characteristics of backfill slurries prepared using PG with different pH values.

Batch no	Viscosity (mPa s)	Bleeding rate (%)	IST (h)	FST (h)
PG-1-O^a^	769	29.13	–	–
PG-1-P^b^-3.50	619	58.00	90	134
PG-1-P-5.00	182	64.21	85	118
PG-2-O	707	33.65	–	–
PG-2-P-3.50	437	68.97	66	74
PG-2-P-5.00	180	73.78	54	66
PG-3-O	490	44.22	100	140
PG-3-P-3.50	404	56.90	78	116
PG-3-P-5.00	116	88.57	65	76
PG-4-O	397	40.21	64	75
PG-5-O	157	58.48	52	74

^a^Original.

^b^Pretreatment.

#### Variation of viscosity in backfill slurry

**Figure 6:**
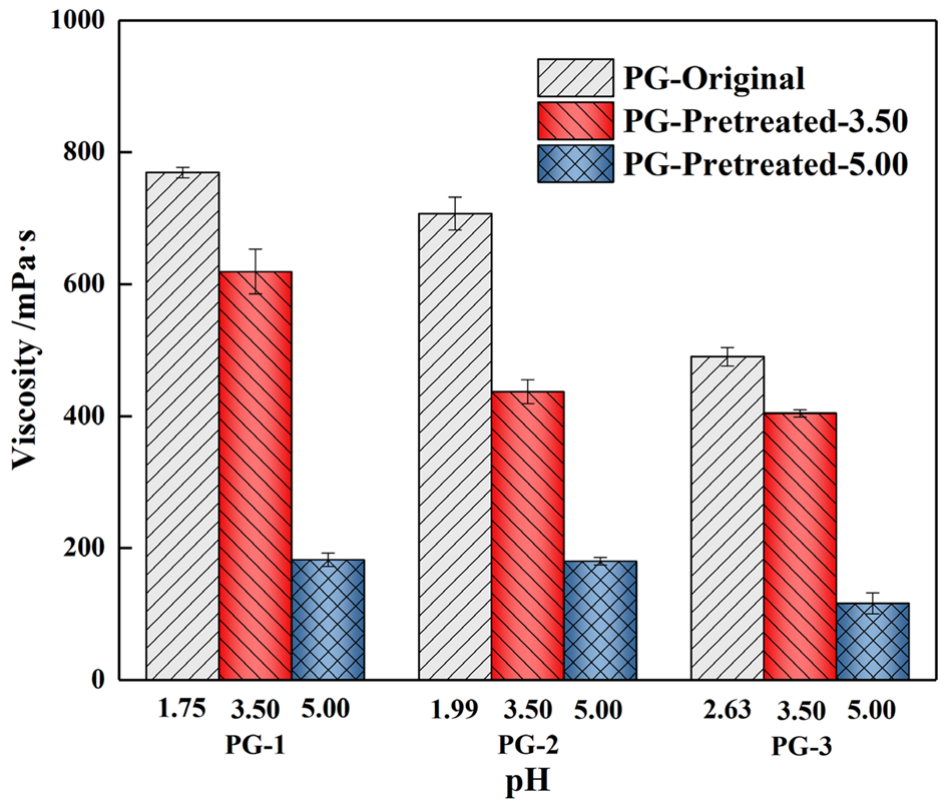
Variation of viscosity in backfill slurry.

In the backfilling process, the slurry is usually mixed on the ground surface and then pumped into the goaf through the pipeline. Excessive viscosity of the slurry may cause a series of problems in slurry mixing, pumping and transportation^32^. Figure 6 shows the variation of slurry viscosity with different pH values of PG. Using original PG as aggregate, the viscosity of backfill slurry decreased from 769·s to 490 mPa·s, with the increase in the pH value of original PG from 1.75 to 2.63. The decrease indicates that the pH value of aggregate has a significant effect on the backfill slurry. As in this study, the viscosity decreased about 75% when the PG (PG-1, PG-2 and PG-3) was washed to a pH value of about 5.00, which was more favorable to the slurry flow^29^. These decreases may be explained by the fact that the surface of PG crystals becomes smooth after the residual acids are washed out, reducing the number of direct crystal-crystal contacts and increasing the thickness of the lubricating film around the crystal^33^. Thereby, the friction force and pressure differential resistance are continuously reduced during the slurry flow process, manifested as a decrease in viscosity.

#### Variation of bleeding rate in backfill slurry

**Figure 7:**
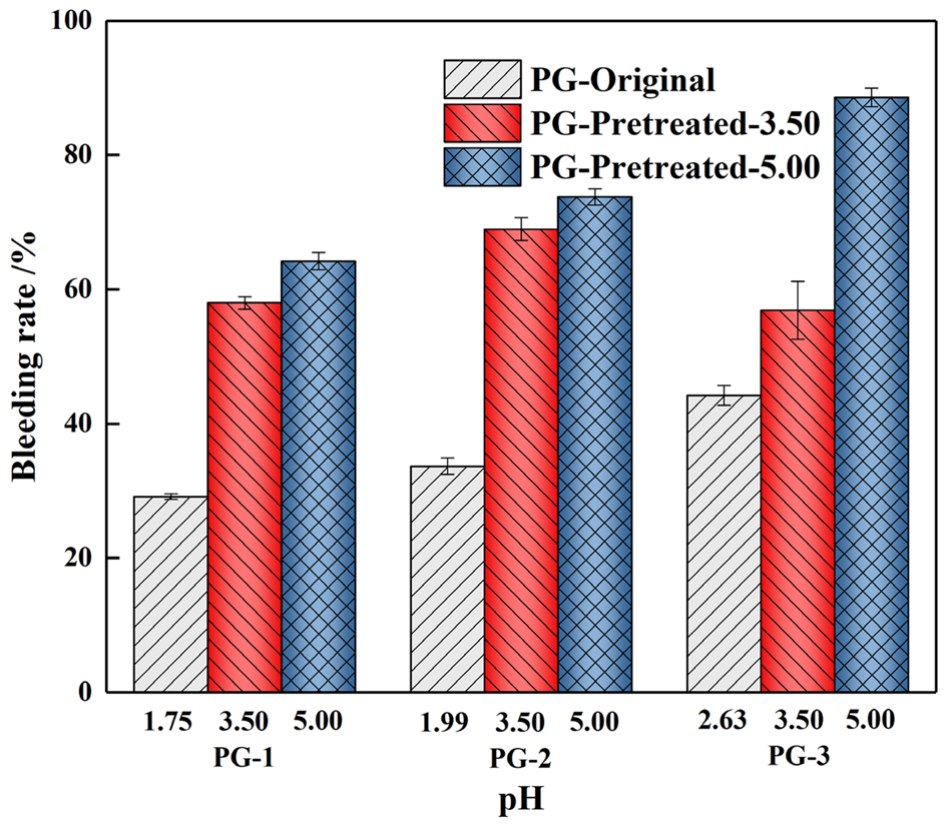
Variation of bleeding rate in backfill slurry.

The bleeding rate affects the durability and strength of the hardened backfill, which is one of the main physical properties^34^. As shown in Fig. 7 and Table 3, the pretreatment of PG significantly affected the bleeding rate of the slurry. With the increase in the pH during the washing process, the bleeding rate of three groups PG-1, PG-2 and PG-3 increased significantly by 120%, 119% and 100%, respectively. This increase was caused by the reduction of residual acids and impurities in PG, which reduced the viscosity of the backfill slurry and weakened the free-water absorption capacity of the slurry. Therefore, the macroscopic performance is the gradual increase in the bleeding rate. For the original PG, as the initial pH value increase from 1.75 to 2.63, the bleeding rate increased by 52%, which is also attributed to this reason. The variation of viscosity and bleeding rate indicates that the water washing pretreatment can effectively improve the fluidity and transportation of the backfill slurry.

#### Variation of setting time in backfill slurry

**Figure 8:**
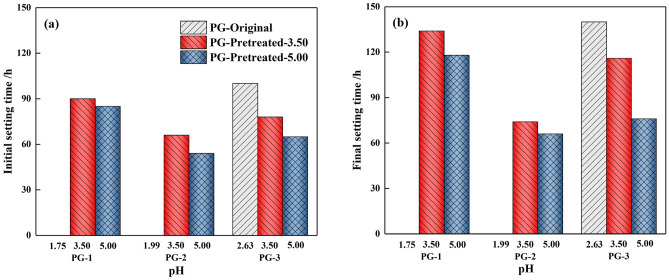
Variation of setting time in backfill slurry: (**a**) IST, (**b**) FST.

The setting time affects the cementation and early strength of the backfill in the backfilling process^35^. The initial setting time (IST) and final setting time (FST) of the backfill slurry are presented in Fig. 8 and Table 3. The slurry prepared from the original PG-1 and PG-2 was not completely set within 7d, so the IST and FST were not measured. A possible explanation for this finding could be that the initial pH value of PG-1 and PG-2 are relatively low. With binder addition into PG, the binder first reacted with PG in a neutralization reaction, consequently slowing down the hydration reaction and prolonging the setting time^31^. For the backfill slurry prepared from pretreated PG, the binder could more rapidly and more extensively participate in the hydration reaction, thus shortening the setting times. It can be evidently seen from the figure that when the pH value of PG-1 and PG-2 was washed to 3.50, the setting times were greatly shortened. As the pH value increased to 5.00, the IST of PG-1 and PG-2 continued to reduce for 5 h and 12 h, and the FST was reduced by 16 h and 8 h, respectively. As the pH value of PG-3 was washed to 5.00, the IST and FST was reduced by 35% and 46%. Overall, the results of setting times can be concluded that the increase in pH value of pretreated PG facilitates the solidification of the backfill slurry into the hardened backfill.

### Strength of pretreated PG-based cemented backfill

**Figure 9:**
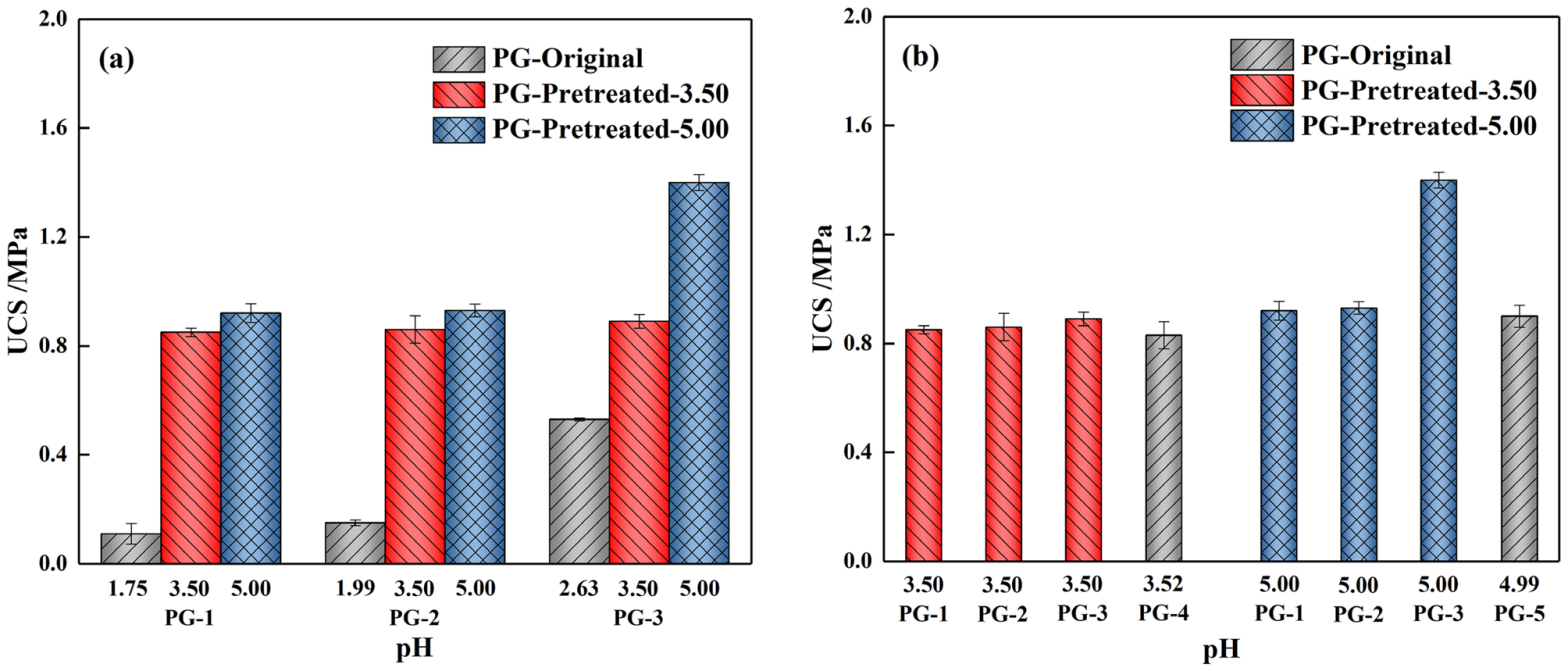
Unconfined compressive strength of 28d backfill prepared by PG with and without pretreatment.

The backfill slurry is pumped into the goaf and then cemented into a hardened backfill with a certain strength, and the strength directly affects the stability of the stope^36^. Studies have shown that the required 28d static strength for cemented backfill without exposure is commonly more than 0.2 MPa^28,37^. Herein, the 28d strength of cemented PG backfill with and without pretreatment was measured, as shown in Fig. 9.

Figure 9a shows that the pretreatment of aggregate PG could well enhance the strength of the backfill. The backfill strength was enhanced significantly by 8.1 times, 6.2 times and 2.7 times by pretreating the three batches of aggregates. This increase in the backfill strength can be explained by the following three reasons. For one thing, it is known that the backfill strength derives from the overlap and tight bonding of aggregate PG and hydration products of the binder^38^. The pretreatment smoothed the PG surface and facilitated the overlap of hydration products and aggregate. For another, PG contains residual acid, which could consume the alkalinity of the binder and reduce the hydration products. As mentioned in 3.4.1, the pH value of the backfill slurry prepared from PG-1-O and PG-2-O are both around 8, causing the 28d strength of hardened backfill to be less than 0.15 MPa. Water washing could remove the majority of residual acids in PG. As the pH value of the pretreated PG reached 3.50 and 5.00, the pH value of the slurry reached around 12.8, which could ensure the proceed of the hydration reaction. The third reason for pretreatment enhancing the backfill strength was the reduction of soluble impurities. The excessive anions in PG would react with Ca^2+^ of the binder, forming insoluble precipitations attached to the hydration products, therefore lowering the quality of hydration products. As shown in Figs. 3 and 5, the content of impurities in PG decreased significantly after pretreatment, so the quality of hydration products improved and backfill strength increased accordingly. In addition, when PG with relatively low initial pH value (1.76, 1.99 and 2.63) was washed to pH 3.50, an evident increase in strength was observed, indicating proper water washing could well enhance the strength development of backfill. However, when the pH value increased from 3.5 to 5.0, a very slight increase was observed for 28d backfill strength. This result indicates that excessive pretreatment of aggregate was unhelpful for backfill strength.

Figure 9b shows the backfill strength prepared from PG washed to the same pH value. Two PG with an initial pH of 3.52 and 4.99 were selected as the control group. When PG with different initial pH was washed to the same pH value, the backfill obtained a similar 28d strength. For example, using PG with a pH value of 3.50 as aggregate (four batches, pretreated or original), the 28 d backfill strength was similar at about 0.9 MPa. This result indicates that pH value could be used as an index to evaluate the quality of PG. To save costs, the degree of water washing should be controlled within a reasonable range based on the actual mining method of the mines.

**Figure 10:**
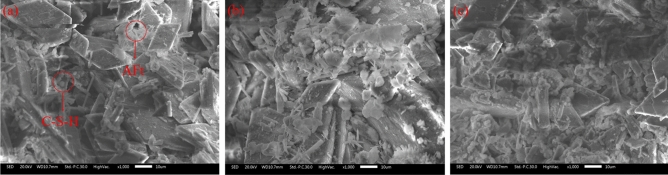
SEM images of cemented PG backfill samples: (**a**) PG-2-O, (**b**) PG-2-P-3.50, (**c**) PG-2-P-5.00.

The SEM images shown in Fig. 10 describe an overview of the microscopic observations of the backfill prepared by PG-2 with and without pretreatment. A large number of exposed plate-like PG crystals can be seen in Fig. 10a, interspersed with a small amount of C–S–H gel and ettringite (AFt). When PG was pretreated, the content of hydration products noticeably increased d (seen in Fig. 10b, c), leading to an increase in strength by 4.7 and 5.2 times. Therefore, the water washing of aggregate can effectively improve the strength of cemented PG backfill.

### Environmental behavior of purified PG in the backfill process

The majority of impurities could be either removed by water washing pretreatment or solidified/stabilized (S/S) by hydration reactions of the binder. However, it remains to be explored whether the bleeding water of backfill slurry and the leaching water of cemented backfill carries unconsolidated impurities and escapes into the groundwater^36^. Therefore, it is necessary to comprehensively understand the environmental behavior of impurities in the backfill process.

#### Impurities in bleeding water

**Figure 11:**
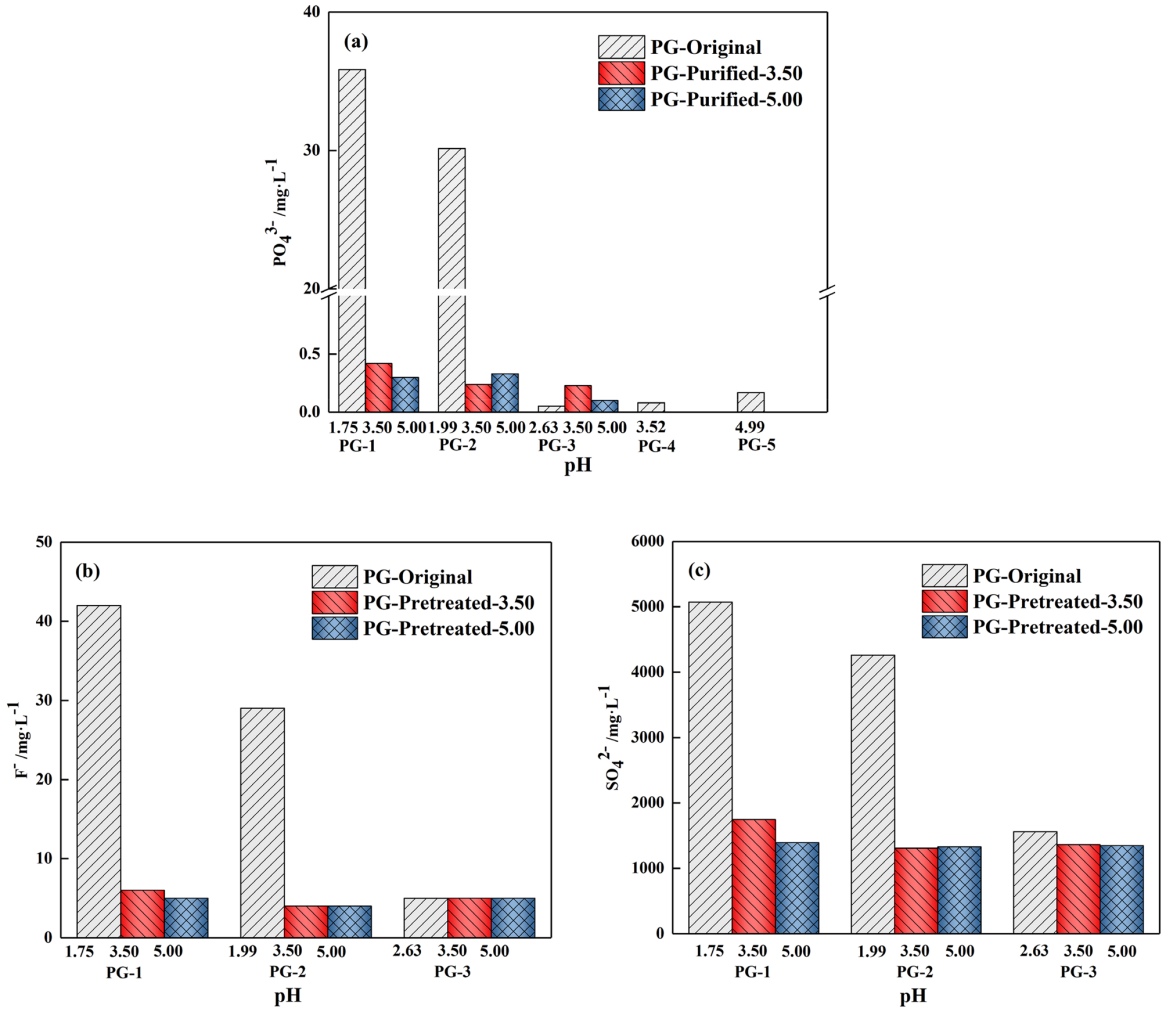
Variation of impurities in bleeding water: (**a**) PO_4_^3−^, (**b**) F^−^, (**c**) SO_4_^2−^.

Figure 11 depicts the concentration of PO_4_^3−^, F^−^ and SO_4_^2−^ in the bleeding water prepared from PG with and without pretreatment. By comparing the PO_4_^3−^ concentration in the PG and the bleeding water, it was found that the hydration reaction was able to consolidate 99% of PO_4_^3−^, which has also been demonstrated by the previous study^31^. However, relatively high PO_4_^3−^ concentrations (35.84 mg/L and 30.15 mg/L) were observed in the bleeding water with PG-1-O and PG-2-O. When the aggregate PG was water washed, the concentration of PO_4_^3−^ in all bleeding water was reduced to less than 0.5 mg/L, as shown in Fig. 11a. As for F^−^, the concentration of F^−^ decreased to 4 ~ 6 mg/L (seen in Fig. 11b) after pretreatment. Further, as can be seen in Fig. 11(c), with the increase of pH value of pretreated PG, the concentration of SO_4_^2−^ in the bleeding water of PG-1 and PG-2 groups gradually decreased from 4000 to 5000 mg/L to about 1300 mg/L. Overall, the water washing of PG could well remove the impurities, leading to lower concentrations of impurities in bleeding water. In addition, it can also be observed that when the pH of PG was washed to about 3.50, the impurities in bleeding water could be maintained at relatively stable levels. Among them, the concentration of PO_4_^3−^ and F^−^ have met the Chinese standard GB8978-1996 for integrated wastewater discharge (F^−^ concentration < 10 mg/L and PO_4_^3−^ < 0.5 mg/L).

#### Impurities in backfill leachate

The toxicity leaching test was conducted on the backfills cured for 28d, and the degree of S/S of impurities in cemented PG backfill was investigated, as shown in Table 4. The PO_4_^3−^ concentrations in all leachate were less than 0.5 mg/L, and the F^−^ concentrations were less than 10 mg/L (except for PG-1-O), which met the Chinese standard for integrated wastewater discharge. The SO_4_^2−^ concentrations were controlled at around 30 mg/L, except for PG-1-O with SO_4_^2−^ concentration up to 104 mg/L. Almost 100% of PO_4_^3−^, more than 99.3% of F^−^ and SO_4_^2−^ in pretreated PG were consolidated. This also proved that the backfill prepared with pretreated PG could significantly alleviate the environmental pollution of PG.

**Table 4:** Experimental data of the backfill leachate.

Batch No	PO_4_^3−^ (mg/L)	F^−^ (mg/L)	SO_4_^2−^ (mg/L)
PG-1-O	0.12	12	104
PG-1-P-3.50	0.05	4	28
PG-1-P-5.00	0.08	4	30
PG-2-O	0.02	8	29
PG-2-P-3.50	0.03	1	32
PG-2-P-5.00	0.09	1	29
PG-3-O	0.03	2	27
PG-3-P-3.50	0.02	2	29
PG-3-P-5.00	0.02	2	26

## Conclusion

The purpose of this study was to investigate the effects of stirring times, S/L ratio and initial pH value of PG on the mechanical properties and environmental behavior of the backfill using the water-washed PG as aggregate. The experimental results show that the pretreatment of aggregate could effectively improve the performance of backfill. The following conclusions can be drawn:

1. The PG pretreatment process was optimized for backfill, including 5 min stirring time, the S/L ratio of 1:0.5.
2. Using pretreated PG as aggregate effectively improved the workability of the backfill slurry and enhanced the strength development of the hardened backfill.
3. Water washing pretreatment significantly reduced the impurities content in the bleeding water and the leachates of backfill. Eventually, almost 100% of PO_4_^3−^, more than 99.3% of F^−^ and SO_4_^2−^ in PG had been fixed into the backfill.
4. The wastewater generated after washing PG could be treated first. For example, by adding the common CaO directly to the wastewater, which is relatively easy to operate. The treated water could still be used to wash PG, realizing the circulation of water resources.
5. In practice, it is recommended to use the pH value of PG as a parameter for selecting the pretreatment method to meet the mechanical and environmental requirements of the backfill in mines.

## Data Availability

The authors declare the availability of the data used in the research to obtain the results reported in the manuscript upon reasonable request.
